# Acquired hemophilia A secondary to SARS-CoV-2 pneumonia: a case report

**DOI:** 10.11613/BM.2022.030801

**Published:** 2022-08-05

**Authors:** Brkić Nikolina, Milić Marija, Bekavac Marija, Marković Maja, Perković Dubravka

**Affiliations:** 1 Department of Transfusion Medicine, General County Hospital Vinkovci, Vinkovci, Croatia; 2 Clinical Institute of Laboratory Diagnostics, University Hospital Osijek, Osijek, Croatia; 3 Faculty of Medicine, University of Osijek, Osijek, Croatia; 4 Department of Hematology, University Hospital Osijek, Osijek, Croatia; 5 Clinical Institute of Transfusion Medicine, University Hospital Osijek, Osijek, Croatia

**Keywords:** hemophilia A, SARS-CoV-2, factor VIII

## Abstract

The acquired hemophilia A (AHA) is a life-threatening condition. The incidence of AHA is extremely low, which requires a multidisciplinary approach to diagnosis and treatment. This is case report of 73-year-old man who presented with AHA secondary to severe acute respiratory syndrome Coronavirus 2 (SARS-CoV-2) pneumonia. The patient had extensive skin bleeding and hematomas. In the coagulation screening tests activated partial thromboplastin time (APTT) was prolonged with normal prothrombin time (PT), which was indication for further investigation. The APTT in a mixing study with normal plasma did not correct so clotting factors inhibitors were suspected. With signs of bleeding, extremely low factor VIII (FVIII) activity (2%) and presence of FVIII inhibitors, AHA was diagnosed and treatment initiated. Patient was treated with factor eight inhibitor bypassing agent (FEIBA) for three days, followed by long-term corticosteroid and cyclophosphamide therapy. Malignant and autoimmune diseases as the most common causes of AHA were ruled out. The patient had a good response to therapy with gradual normalization of APTT and FVIII activity. To the best of our knowledge, the present case is the first reported case of *de novo* AHA after SARS-CoV-2 pneumonia. The diagnosis of AHA should be suspected in a patient with bleeding into the skin and mucous membranes without a previous personal and family history of bleeding, and with isolated prolonged APTT. It is important to investigate any isolated prolongation of APTT in cooperation with clinical laboratory experts.

## Introduction

The acquired hemophilia A (AHA) is a life-threatening condition. The incidence is extremely low (1-4 *per* million inhabitants/year), which requires a multidisciplinary approach to diagnosis and treatment (*1*).

The acquired hemophilia A occurs in patients with no medical history of bleeding disorders. The disorder is caused by autoantibodies to factor VIII (FVIII). Often patients with AHA have a malignant or autoimmune disease in the background, but the cause for about 50% of cases is still unclear (*2*). Most patients with AHA have signs of bleeding on the skin and/or mucous membranes. Bleeding can be lethal in 20% of all symptomatic cases (*2*). Unlike congenital hemophilia, bleeding into the joints is rare.

In coagulation screening tests, patients with AHA have prolonged activated partial thromboplastin time (APTT) and normal prothrombin time (PT), which requires further laboratory investigation. Communication between the clinician and the clinical laboratory expert is important to clarify the causes of prolonged APTT, to select additional tests and to interpret the laboratory findings.

To the best of our knowledge, the present case is the first reported case of *de novo* AHA after severe acute respiratory syndrome Coronavirus 2 (SARS-CoV-2) pneumonia. Since there is growing evidence associating COVID-19 infection with autoimmune disease, for example, autoimmune hemolytic anemia, thrombotic thrombocytopenic purpura and immune thrombocytopenia, we found important to report this case in order to collect as much data as possible about immunologic complications of Coronavirus Disease 2019 (COVID-19) (*3*-*5*).

## Case report

Written informed consent was obtained from the patient. A 73-year-old man was hospitalized at the General County Hospital from January 23 to February 2, 2021, under diagnosis of bilateral pneumonia caused by SARS-CoV-2 virus, confirmed by PCR test. His comorbidities included type 2 diabetes, arterial hypertension, Arnold-Chiari syndrome, and prostate hyperplasia. He had no bleeding disorders during his lifetime. Regular chronic therapy before hospitalization was atorvastatin, metformin and tamsulosin.

At admission, patient presented with normocytic anemia, increased liver enzymes, normal PT, and prolonged APTT. Markers of kidney function were within the reference interval (RI). The values of the parameters are shown in Table 1.

**Table 1:** Laboratory findings from General Hospital

**Date/** **Parameters**	**23.01.** **2021.**	**02.02.** **2021.**	**06.03.** **2021.**	**08.03.** **2021.**	**15.03.** **2021.**
Hb (g/L, RI 138-175)	105	101	116	120	125
Plt (x10^9^/L, RI 158-424)	236	518	288	240	238
AST (U/L, RI 11-38)	195	87	23	NT	19
ALT (U/L, RI 12-48)	197	249	22	NT	18
GGT (U/L, RI 11-55)	358	245	NT	NT	NT
PT (%, RI > 70)	133	NT	115	115	123
APTT (s, RI 22-32)	56.0	NT	NT	44.5	47.0
APTT R (RI 0.8-1.2)	2.08	NT	NT	1.66	1.74
TT (s, RI 14-21)	13.8	NT	NT	13.3	16.6
HB – hemoglobin. RI - reference interval. Plt – platelets. AST - aspartate aminotransferase. ALT - alanine aminotransferase GGT - gama-glutamyl transpeptidase. PT - prothrombin time. APTT - activated partial thromboplastin time. R – ratio. TT - thrombin time. NT - not tested.					

During hospitalization he received nadroparin in prophylactic dose, ceftriaxone, dexamethasone, and proton pump inhibitor. In medical documentation it is noted that there were no signs of rash or bleeding on the skin or mucous. Patient was released after 11 days of treatment in an improved condition, with his chronic therapy. Laboratory findings at the day of dismission are presented in Table 1.

On March 6th (a month after hospitalization), patient was examined at the emergency room for swelling of both lower legs. Suffusions of the skin of the right forearm, under the left armpit and on the left leg were described. In the coagulation screening test PT was normal and D-dimer elevated (1600 µg/L FEU). Other laboratory findings are shown in Table 1. Color doppler of the legs showed no signs of deep vein thrombosis. The patient was advised to take enoxaparin in a prophylactic dose for 3 weeks. Two days after (on March 8th), he came for spontaneous hematomas to the Coagulation disorder clinic. Laboratory findings are presented in Table 1. As APTT was still prolonged, it was recommended to stop enoxaparin therapy, and to come for a check-up in a week.

At the follow-up examination on March 15th, he had a few skin suffusions (up to 2 cm in diameter) on the left thigh, in the left armpit, on the right forearm - all were in regression, without new changes. Prophylactic therapy with low molecular weight heparin (LMWH) lasted only 2 days so the possible effect of heparin on APTT was ruled out. Liver enzymes and renal function were in the RI, and the cause of the bleeding was not clear. Further research into the causes of prolonged APTT was agreed during communication with a specialist in the clinical laboratory.

On April 29th, the patient was hospitalized at the Hematology department of the University Hospital. He had clinically significant signs of bleeding: hematoma occupied almost the entire inner side of the right forearm, suffusion in the medial part of the right thigh was about 15x12 cm. Coagulation screening tests revealed isolated prolonged APTT (Table 2) that was not corrected in a mixing study. Mixing test was performed as follows: patient plasma was mixed with normal plasma in ratio 1:1. Activated partial thromboplastin time was measured immediately and after 2 hours of incubation at 37 °C. It remained prolonged upon incubation suggesting the presence of a time dependent inhibitor. Reagent (Actin FS, Siemens) that has a very low sensitivity for lupus anticoagulant (LA) was used to measure APTT. The activities of all intrinsic pathway coagulation factors were measured by clot-based method. Factor activities were slightly below RI (F IX 51% (RI 55-163), F XI 57% (RI 67-127), F XII 45% (RI 49-141) except for FVIII which was < 2%. Factor XIII and Von Willebrand factor were normal. Factor VIII activity was measured in several dilutions of patient plasma with buffer which did not lead to normalization of FVIII activity. Due to the very low activity of factor VIII, presence for FVIII inhibitors was tested as follows. The residual FVIII activity (3%) was calculated from the FVIII activity in the test mixture (containing patient plasma and a normal plasma) and control mixture (containing FVIII deficient plasma and normal plasma).

**Table 2:** Presentation of coagulation screening test results during control examinations in the University Hospital

**Date/** **Parameters**	**29.04.** **2021.**	**19.05.** **2021.**	**31.05.** **2021.**	**11.06.** **2021.**	**21.06.** **2021.**	**02.07.** **2021.**	**08.07. 2021.**	**09.09.** **2021.**	**11.11.** **2021.**
PT(%, RI > 70)	137	117	130	131	131	131	131	126	126
TT (s, RI 14-21)	15.5	NT	16.4	16.4	16	16.2	14.8	15.1	16.2
APTT ratio (RI 0.80-1.20)	1.75	1.65	1.37	1.28	1.24	1.22	1.15	1.17	1.23
APTT (s, RI 22-32)	47.3	44.5	37.0	34.6	33.5	32.9	31.1	31.6	33.2
Fibrinogen (g/L, RI 1.8-3.5)	3.9	2.4	3.0	3.9	NT	4.1	5.4	4.4	3.5
FVIII activity (%; RI 50-149)	2	< 2	3	8	6	15	22	54	30
PT - protrombin time. RI - reference interval. TT - thrombin time. APTT - activated partial thromboplastin time. NT - not tested.									

Since LA may also be the cause of prolonged APTT, presence of LA was tested and was weakly positive. Lupus anticoagulant testing was performed using Diluted Russell Viper Venom Time test (dRVVT) and LA-sensitive APTT in three steps (screening, mixing and confirmatory). While dRVVT was negative, all three steps for LA-sensitive APTT tests were weakly positive. Antiphospholipid antibodies were negative (anticardiolipin IgG and IgM, and beta-2 glycoprotein). Anti-SS-A/Ro was positive, and titer of anti-nuclear factor (ANF) was 1:100. That findings are a possible consequence of SARS-Cov-2 infection because there were no clinical signs of immunological disease.

Malignant disease was excluded (ultrasound of abdomen, computed tomography of abdomen, chest X-ray, positron emission tomography/ computed tomography of all body). Cytokeratin fragment 21-1 was mildly elevated (3.8 µg/L, RI > 3.3), with other tumor markers in the RI (carbohydrate antigen 19-9, carcinoembryonic antigen, alpha fetoprotein, prostate specific antigen, neuron-specific enolase).

The patient was treated with factor eight inhibitor bypassing agent (FEIBA) for three days, followed by continued methylprednisolone and cyclophosphamide therapy. He was hospitalized for 35 days (from April 30th to June 4th). Therapy for discharge was methylprednisolone 32 + 16 mg, cyclophosphamide 3 tablets of 50 mg, with other chronic therapy rosuvastatin, tamsulosin, pantoprazole, gliclazide, sitagliptin and metformin.

Follow-up examinations were every 2 weeks. The findings of coagulation screening tests and FVIII activity are presented chronologically in Table 2. In repeated testing (3 months after initial testing) LA was negative. The patient had complications of AHA treatment on two occasions. The first complication was leukopenia due to cyclophosphamide administration. Methylprednisolone was later ruled out because it most likely caused myopathy and leg pain.

After 4 months of intensive treatment and follow-up, the patient had no new signs of bleeding. The patient is still under the control of a hematologist and on cyclophosphamide therapy. The last check-up was done on March 24th, 2022. In the laboratory findings APTT is in RI and FVIII activity is 30%.

## Discussion

The patient presented here, had a significantly prolonged APTT on his first admission due to SARS-CoV-2 pneumonia (in January 2021). No additional coagulation tests were performed because he had no signs of bleeding. A possible explanation for the fact that the patient had no signs of bleeding during first hospitalization is dexamethasone treatment of SARS-CoV-2 infection. When the bleeding occurred the diagnosis of AHA was further complicated since the patient was taking enoxaparin therapy. Therefore, the diagnosis of AHA was postponed. Delays in diagnosis together with the severity of bleeding, are concurrent causes of the reported high rate of mortality (up to 41%) associated with AHA (*6*). Therefore, it is important to quickly diagnose AHA.

Key clinical picture in AHA is bleeding on the skin and mucous membranes. Other possible causes of bleeding (liver damage, disseminated intravascular coagulopathy (DIC), anticoagulant therapy, *etc.*) must be ruled out. In patient presented here, transient elevated liver enzymes were understood as part of the clinical picture of COVID-19, and there were no other signs of liver damage. Normal PT also indicated normal liver function. Disseminated intravascular coagulopathy was ruled out because the patient was not in sepsis, and there were no other DIC trigger and no signs of internal organ microthrombosis. The findings of laboratory parameters that are disturbed in DIC (platelet count, PT, thrombin time (TT), and fibrinogen) were within RI.

For the diagnosis of AHA the key coagulation laboratory finding is a prolonged APTT with a normal PT. Communication between the clinician and the clinical laboratory expert is important to clarify the causes of prolonged APTT, to select additional tests and to interpret the laboratory findings. In patient presented here effect of LMWH on prolonged APTT was ruled out because a month after cessation of LMWH therapy patient developed bleeding symptoms. Besides, for prophylactic doses of LMWH, no significant effect on APTT is expected. Contamination of the sample with unfractionated heparin was also ruled out since TT was normal and prolonged APTT was found on several occasions. The APTT remains prolonged in the mixing test of patient’s plasma with normal plasma, indicating the presence of clotting factors inhibitors. Factor VIII activity is reduced and the FVIII inhibitors are detected. At the same time presence of LA must be excluded. Laboratory distinction between an LA and intrinsic factor inhibitor is difficult since LA can interfere with phospholipid-dependent APTT assays and *vice versa*. In our case, APTT reagent that has a very low sensitivity for LA was used in routine screening and factor activity measurement to minimize effect of LA on these tests. The APTT mixing study results suggested a time dependent inhibitor, which is more typical of FVIII inhibitors than LA, further supporting the presence of a FVIII inhibitors. Factor VIII activity was measured in several dilutions to exclude LA inhibitor effect (nonparalelism). Based on all these results and the clinical picture (bleeding diathesis) of the patient, the diagnosis of AHA was made. The limitation of this case report is that the titer of FVIII inhibitor was not measured as it was not available.

The first case of AHA after SARS-CoV-2 infection was presented by Franchini *et al*. (*6*). The patient had bilateral interstitial pneumonia and hematoma in the trunk, with prolonged APTT. However, this was a relapse of AHA since the patient was diagnosed with AHA in 2011. Olsen *et al.* described an 83-year-old woman with *de novo* AHA after asymptomatic SARS-CoV-2 infection (only ageusia) and no underlying disease (*9*).

A case report by Ghafouri *et al.* presented an 89-year-old patient that was SARS-CoV-2 positive at PCR, with no symptoms of infection (*7*). He had advanced prostatic cancer, hemolytic anemia, positive LA, indeterminately high anticardiolipin IgG antibody and very high FVIII inhibitors (2222 Bethesda units). Besides low FVIII activity, activities of FIX and FXI were also < 1%. One week after hospitalization, the patient developed respiratory failure, DIC, and died of acute cardiopulmonary failure. The only hemorrhagic complication was hematuria.

Wang *et al.* reported an 65-year-old man with autoimmune thyroiditis and hepatitis B infection in medical history (*8*). He presented with severe bleeding in his arm complicated with compartment syndrome. Based on the positive antibodies to SARS-CoV-2, the authors concluded that AHA was secondary to an asymptomatic SARS-CoV-2 infection.

The patients presented by Ghafouri *et al.,* and Wang *et al.*, had other possible causes of AHA (prostatic cancer and autoimmune thyroid disease), while our patient had neither malignant nor autoimmune disease (*7*, *8*).

Hafzah *et al.* reported a case of AHA which developed four months after mild SARS-CoV-2 infection complicated by a pulmonary embolism (*9*). Spontaneous ecchymoses and hematomas in the skin were initially thought to be related to the use of the direct oral anticoagulant apixaban and aspirin. In our case, too, heparin was initially thought to be the cause of the bleeding.

Guerra *et al.* presented 74-year-old female with a gross hematuria and history of recent mild infection with SARS-CoV-2 (*10*). Malignant disease has been ruled out, but the exclusion of autoimmune disease was not reported although she had fibromyalgia in medical history. Nardella *et al.* showed a 53-year-old woman treated under the diagnosis of AHA (*11*). They concluded that AHA may have been associated with SARS-CoV-2 infection based on SARS-CoV-2 anti-spike antibodies, although she also had signs and symptoms of autoimmune thyroiditis.

According to all, the present case is the first reported case of *de novo* AHA secondary to SARS-CoV-2 pneumonia, with malignant or autoimmune disease excluded.

During SARS-CoV-2 infection, hemostatic system disorder is common in terms of a prothrombotic state with a high incidence of deep vein thrombosis and stroke. To clarify the prothrombotic status, several authors examined the presence of antiphospholipid antibodies in SARS-CoV-2 infection, and found a high proportion of patients positive for antiphospholipid antibodies (*12*, *13*). Lupus anticoagulant was transiently positive as it is often in a variety of infections and as was reported for COVID-19 patients (*12*, *13*). Also, it is possible that the FVIII inhibitors interfered in the test to detect the presence of LA (*13*). In our case antiphospholipid antibodies were negative (anticardiolipin IgG and IgM, and beta-2 glycoprotein) which is in accordance with findings of Devreese *et al.* (*13*). They found that the triple antiphospholipid antibodies positivity is rare.

Besides antiphospholipid antibodies development, other immune system disorders in SARS-CoV-2 infection have been described in the literature - autoimmune hemolytic anemia, immune thrombocytopenic purpura, thrombotic thrombocytopenic purpura and Guillain-Barré syndrome (*3*, *4*, *14*, *15*). We believe that the inhibitor to FVIII developed due to immune dysregulation from the underlying COVID-19. Some authors consider that antibodies targeting spike protein are responsible for the development of autoimmunity after SARS-CoV-2 infection because similar immunological reactions occur after vaccination against SARS-CoV-2, and cross-reactivity has been demonstrated *in vitro* with a number of human tissue antigens (*5*, *16*, *17*). Ten cases of AHA after SARS-CoV-2 vaccination have been published so far (*16*-*24*).

Given that the incidence of AHA is very low (1-4 *per* million inhabitants *per* year), and that in the diagnosis beside clinical signs of bleeding, coagulation screening tests are extremely important, we wanted to increase awareness among health care providers about AHA as possible complication of SARS-CoV-2 infection. Under normal circumstances seriously ill patients are treated by hematologists and intensivists, but in pandemic conditions this was not always possible. Often, COVID-19 patients were treated by other specialists due to lack of personnel that are not experts in the management of coagulation disorders and are not skilled in APTT interpretation. So, it is possible that the incidence of AHA secondary to COVID-19 is higher but unrecognized.

Overall, this single case report only suggests a possible association between AHA and SARS-CoV-2 infection. Investigation into the incidence of AHA before and after this pandemic is needed to reveal this association. Also, extensive research is needed to define mechanisms underlying immunologic complications of COVID-19.

## Conclusions

Due to the growing number of reported cases of autoimmune disorders within and after COVID-19, it is possible that AHA is one of the complications of COVID-19. The diagnosis of AHA should be suspected in a patient with bleeding into the skin and/or mucous membranes without a previous personal and family history of bleeding, and with isolated prolonged APTT. It is important to investigate any isolated prolongation of APTT in cooperation with clinical laboratory experts.
